# A Rare Case Report on Xanthogranulomatous Osteomyelitis of Hip Mimicking Tuberculosis and Review of Literature

**DOI:** 10.7759/cureus.5921

**Published:** 2019-10-16

**Authors:** Subodh Pathak, Rakesh Gautam, Prince PC, Priyank Bagtharia, Aryan Sharma

**Affiliations:** 1 Orthopedics, Maharishi Markandeshwar Institute of Medical Sciences and Research, Maharishi Markandeshwar University, Ambala, IND; 2 Orthopedics, Maharishi Markandeshwar University, Ambala, IND

**Keywords:** xanthogranulomatous inflammation, tuberculosis hip, infection, tumor, foamy macrophages

## Abstract

Xanthogranulomatous osteomyelitis (XO) is a rare chronic inflammatory process characterized by the presence of a large number of lipid-containing macrophages with lymphocytes and plasma cells. We present a case of XO of the hip in a 50-year-old woman with pain in the left hip for 28 months. The patient had a history of taking anti-tuberculosis chemotherapy for five months. Laboratory data revealed an increased erythrocyte sedimentation rate and C-reactive protein (CRP) level. Plain radiographs showed the destruction of the femoral head with arthritis and subluxation. Magnetic resonance imaging (MRI) was suggestive of tubercular infection of the left hip and a benign lesion in the left ilium. The histopathologic examination of the specimen demonstrated the presence of dead bone surrounded by lymph-plasma cells, foamy cells, and histocytes, which was consistent with XO, and culture was positive for *Staphylococcus aureus* infection. The patient was successfully treated with resection arthroplasty and antibiotics. It is important for the surgeons to keep XO in the list of differentials in cases with lytic lesions of bone and assessment should include microbiological culture along with the biopsy.

## Introduction

Xanthogranulomatous osteomyelitis (XO) is a rare form of chronic osteomyelitis characterized by the collection of foamy macrophages along with mononuclear cells in the tissue [1]. There have been case reports of xanthogranulomatous inflammation in organs such as gall bladder, kidney, pancreas, fallopian tube, ovary, epididymis, testis, and prostate and salivary glands [2]. But its occurrence in the brain, lungs, and bones is very rare [3]. The radiologic picture almost always has no role in diagnosis as it may show lytic or even blastic lesions with rare periosteal reaction, which mimics a tumor. The challenges faced in diagnosing this condition are to such an extent that all the reported cases were diagnosed only after the biopsy. To our knowledge, this is the first case of XO to be reported presenting as gross hip joint destruction as in tuberculosis of hip joint.

## Case presentation

A 50-year-old woman presented with complaints of pain in the left hip and difficulty in walking for a duration of 28 months. She had intermittent episodes of fever for the last seven months, but no weight loss and other systemic signs. On examining the patient, there was anterior hip joint tenderness with no local signs of any infective or inflammatory pathology. The patient presented with restriction of left hip movements. The patient had received anti-tuberculosis (TB) chemotherapy with isoniazid, rifampicin, pyrazinamide, and ethambutol for around five months in the past (stopped a month before presenting to us). The patient was not ambulatory and was dependent for day-to-day activities.

**Figure 1:**
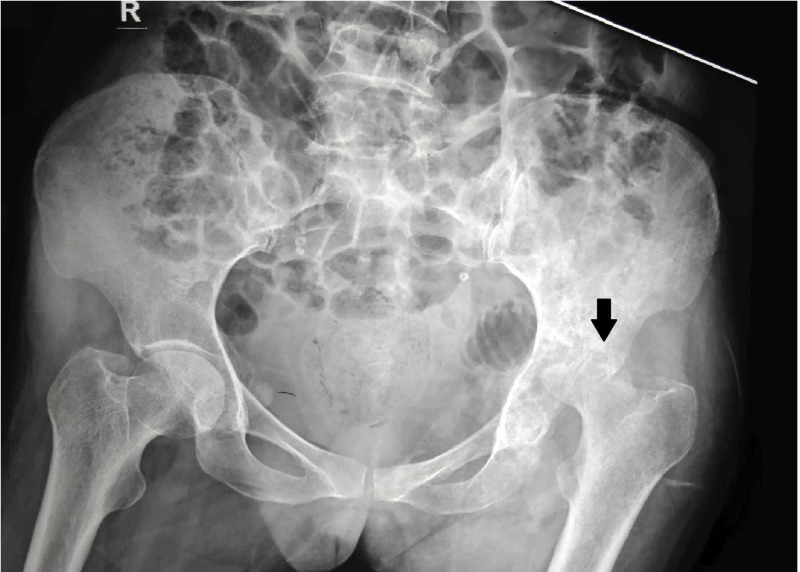
Plain anteroposterior radiographs of pelvis with both hips with the gross destruction of the femoral head with head subluxation (black arrow)

On plain radiographs of the pelvis with bilateral hips, gross destruction of left femoral head and acetabulum with femoral head subluxation was observed (Figure 1).

**Figure 2:**
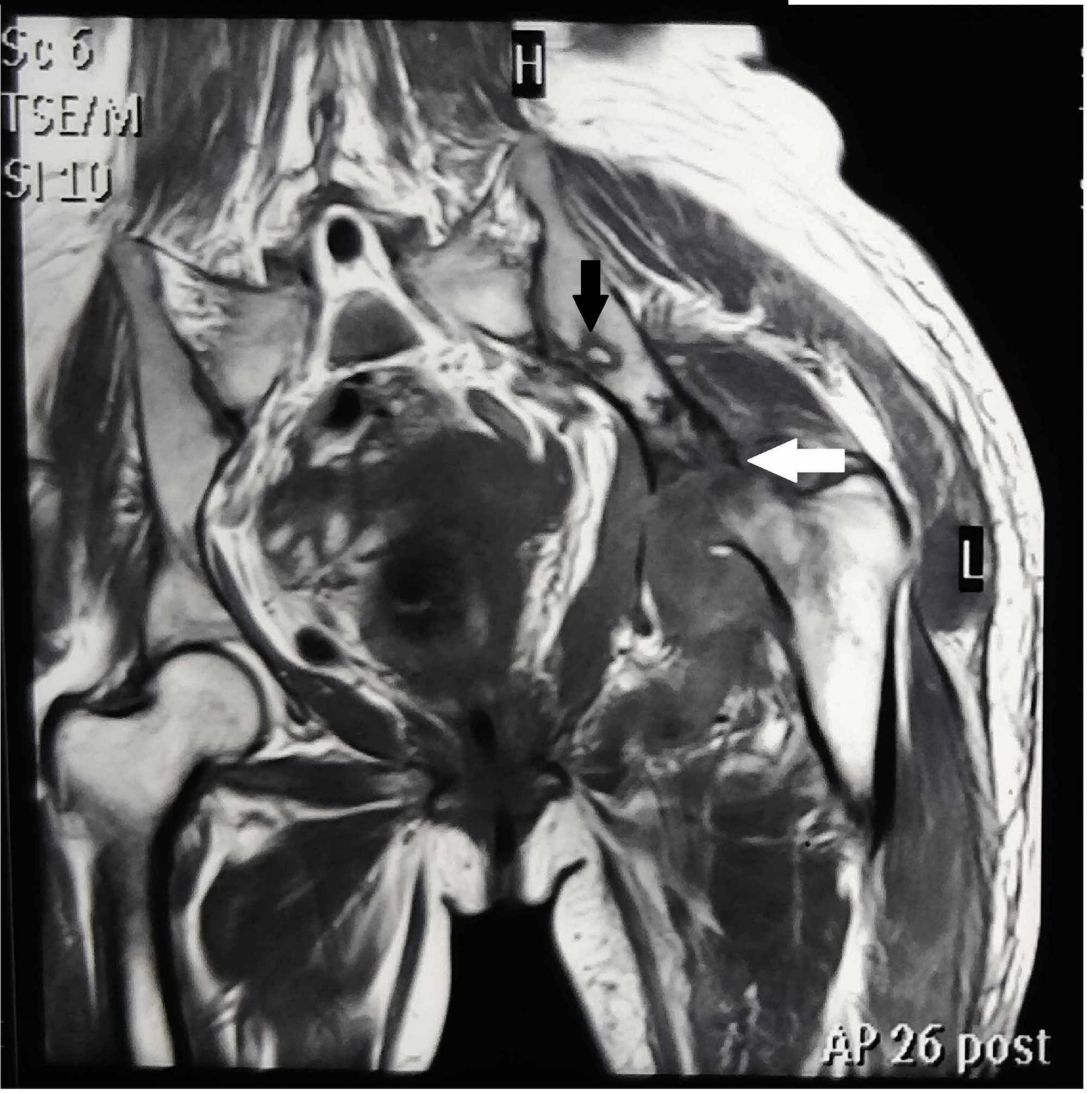
Sagittal T1-weighted MRI image of left hip showing destruction of the left femoral head with superolateral hip dislocation (white arrow) and hypointense focal lesion of the left ilium (black arrow) MRI, magnetic resonance imaging

The inflammatory markers were raised with an erythrocyte sedimentation rate (ESR) of 52 mm/hour (normal, 0-20 mm/h) and C-reactive protein (CRP) level of 27.3 mg/L (normal, 0-3 mg/L), and the leukocyte count was 13,280/mm^3^ (normal, 4400-11,300/mm^3^), with a predominance of lymphocytes. Considering the nature of the lesion radiologically and clinically, a list of differential diagnoses including tubercular osteomyelitis of the left hip, bacterial septic arthritis, and osteoarthritis secondary to avascular necrosis was considered. Hematologic investigations and magnetic resonance imaging (MRI) of the bilateral hips were performed to rule out the differential diagnoses. MRI evidenced synovial thickening of the left hip with superolateral subluxation, extensive erosions along the femoral and acetabular margins, and a focal osseous lesion measuring 11 x 8 mm in the left ilium (Figure 2).

**Figure 3:**
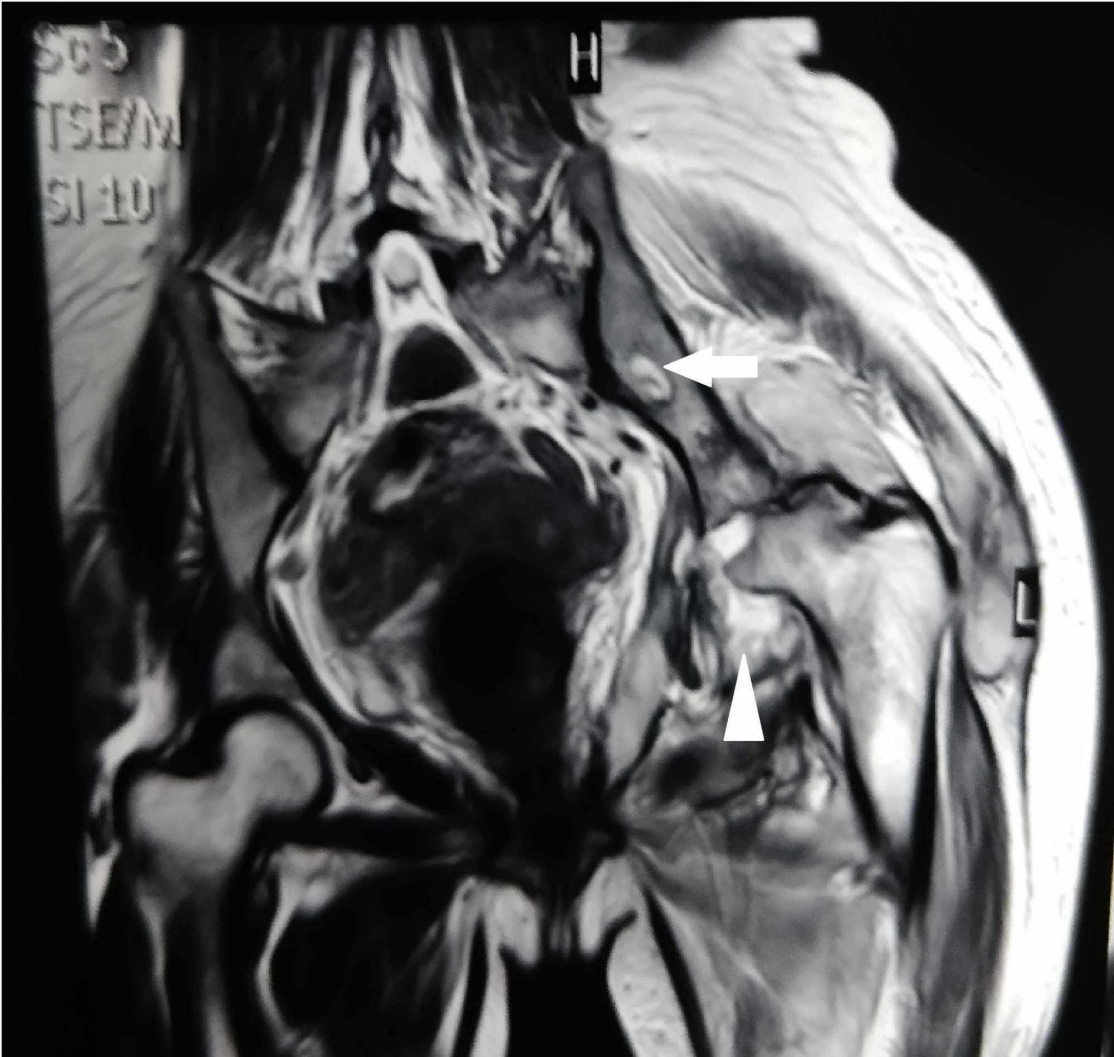
Sagittal T2-weighted MRI showing fluid collection around left hip (white arrow head) with a hyperintense focal osseous lesion in the left ilium (white arrow) MRI, magnetic resonance imaging

Marrow edema extended to the intertrochanteric region. T2-weighted images revealed the fluid level in the anterolateral aspect of the left hip and edema surrounding the hip joint (Figure 3).

The MRI diagnosed it as infective pathology, suggestive of tuberculosis and a benign lesion of the ilium.

**Figure 4:**
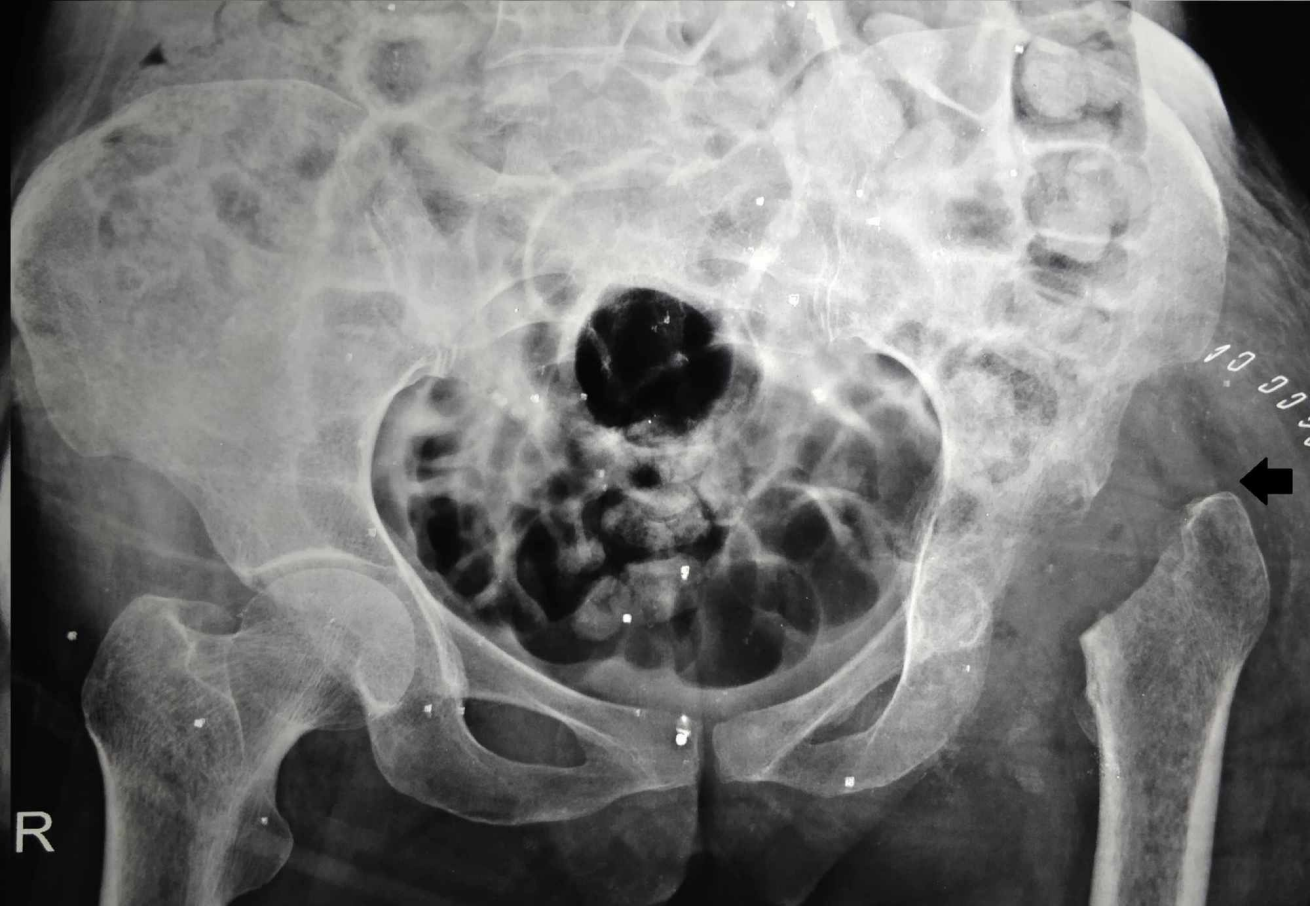
Plain anteroposterior radiographs of pelvis with left femoral head and neck resection and proximal migration of shaft (black arrow)

The patient was taken up for surgery under spinal anesthesia. The standard posterior approach to the hip was used and surgical debridement was done. During surgery, gross destruction of the acetabulum and femoral head with blood and purulent-appearing material were noted. Girdlestone hip arthroplasty was performed (Figure 4), and curettage of lesion of the left ilium was done and tissues were sent for histopathology and culture.

**Figure 5:**
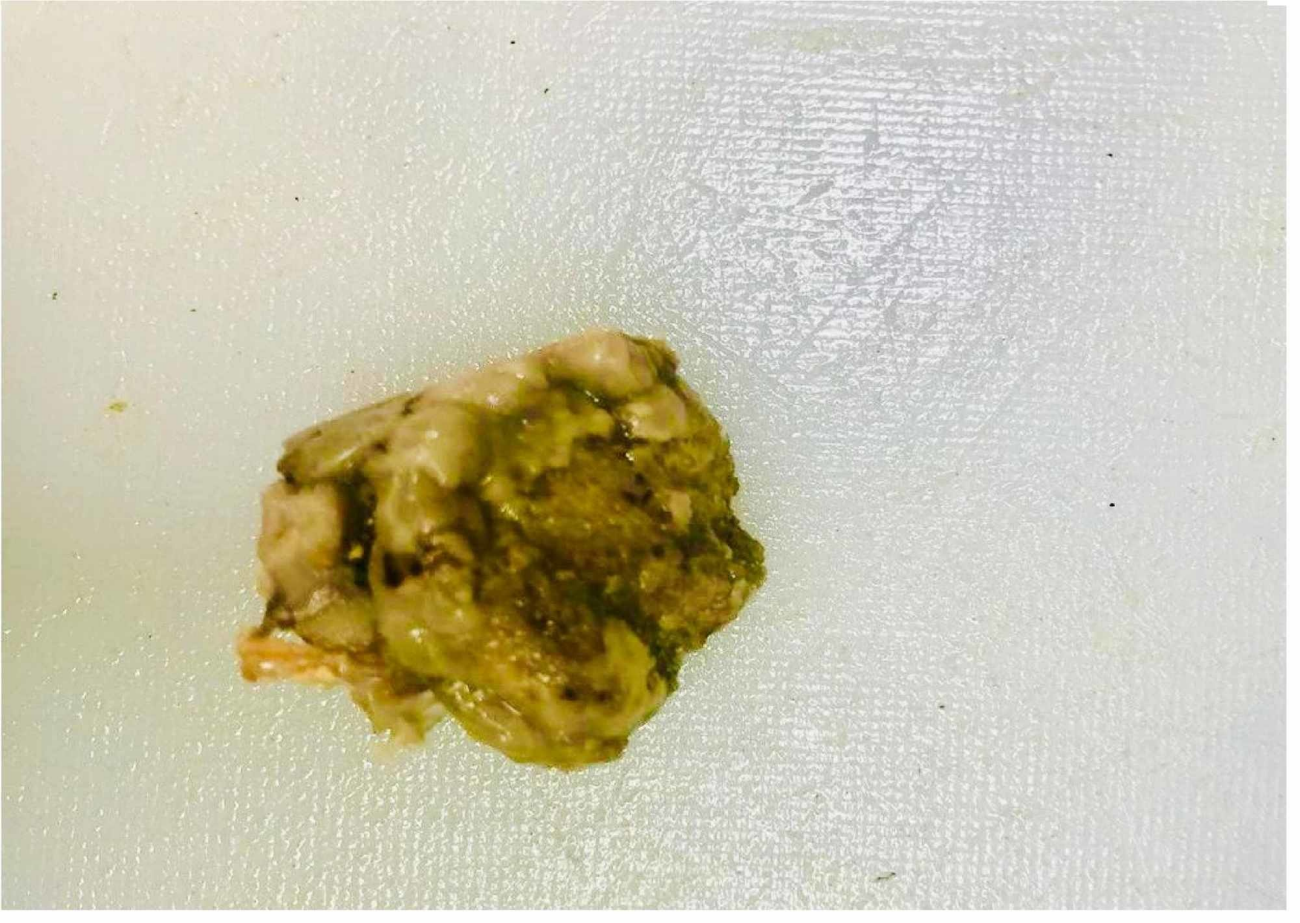
Histopathology sample of resected femoral head

The postoperative period was uneventful. The resected specimen showed a deformed femoral head with severe cartilage destruction (Figure 5).

**Figure 6:**
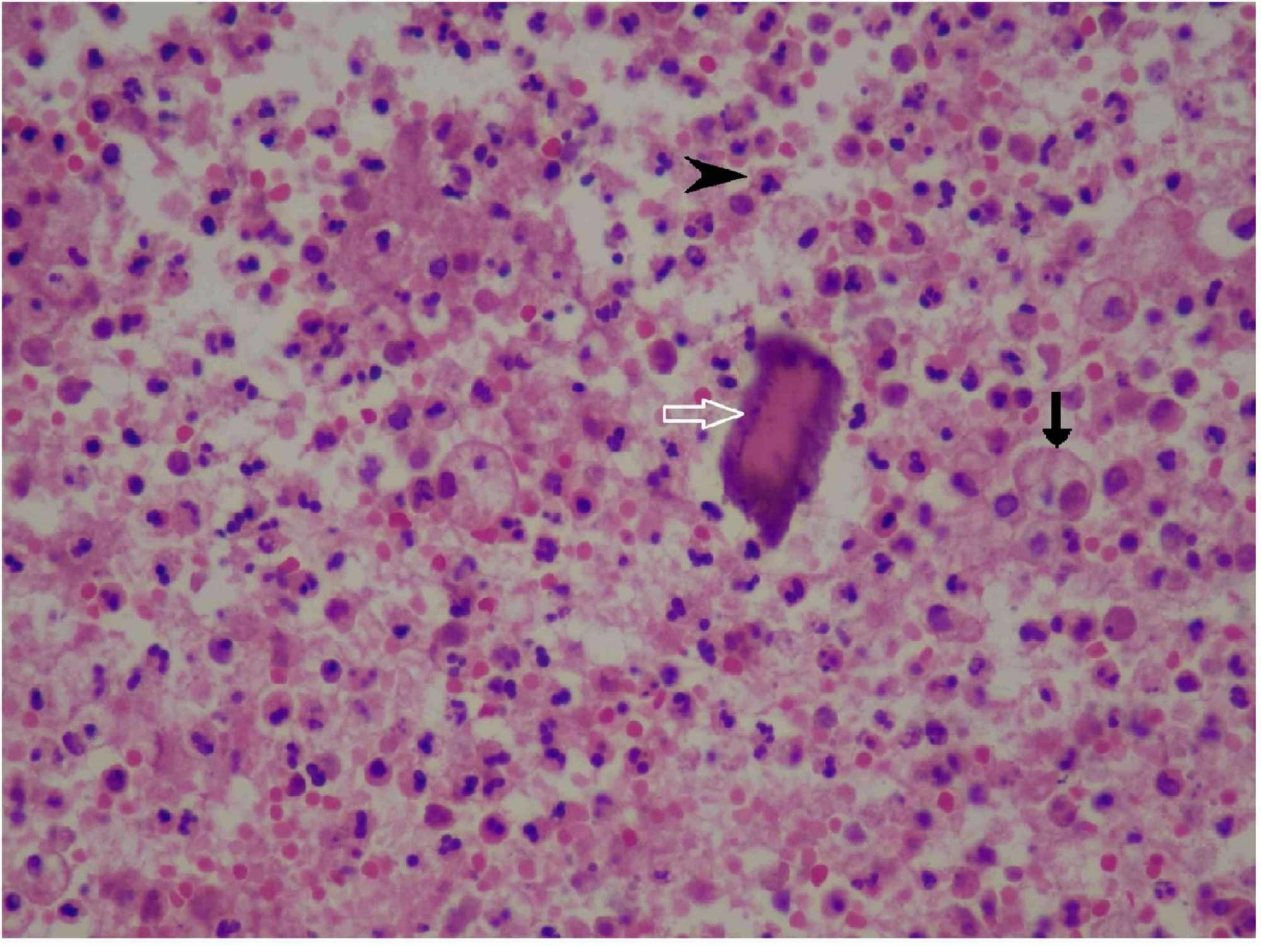
Composite photomicrograph image showing bony spicule with foamy macrophage (hematoxylin and eosin stain, 100x magnification) Black arrow: foamy histiocyte; white arrow: dead bony spicule; black arrowhead: acute inflammatory infiltrate

**Figure 7:**
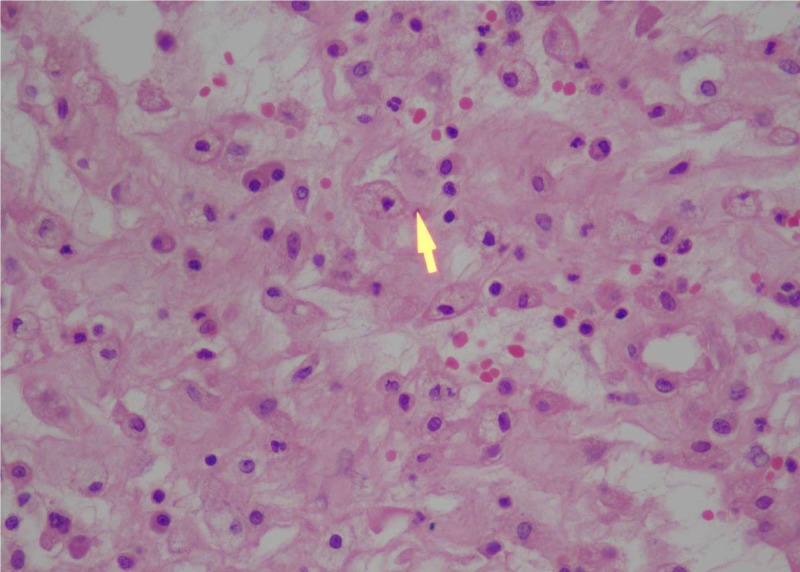
Composite photomicrograph image showing foamy macrophages histiocytes, and plasma cells (hematoxylin and eosin stain, 100x magnification) Yellow arrow: foamy macrophages

Hematoxylin and eosin-stained permanent sections from both sites showed diffuse inflammatory infiltration containing neutrophils and exuberant lymph-plasma cells admixed with foamy macrophages with dead bone and suggestive of XO (Figures 6-7).

 

There was no evidence of malignancy or granuloma. Ziehl-Neelsen stain for tissues of acid-fast Bacilli was negative. Microbiological culture revealed the growth of *Staphylococcus aureus* after 48 hours of aerobic incubation. The patient was started on intravenous (IV) cefuroxime 1.5 g and oral linezolid 600 mg for two weeks. All the antibiotics were started as per minimum inhibitory concentration (MIC) values. During hospitalization, the patient’s general condition improved. The inflammatory markers reduced gradually (CRP level of 6.2 mg/L and ESR of 26 mm/hour) over two weeks. The patient was discharged on oral clindamycin for two weeks. Regular follow-up was done in outpatient with clinical, hematological, and radiological parameters. The ESR was 12 mm/hour and the leukocyte count was 8,120/mm^3^ at follow-up of six months. At 22-month follow-up, the patient had leg length discrepancy of 3.5 cm and displayed a positive Trendelenburg sign and was able to ambulate with aid.

## Discussion

To our knowledge, only a few cases of XO in bone have been reported in the literature. Among these, the earliest two cases were reported by Cozzutto et al., which were in the first rib and the epiphysis of the tibia [3]. A review of cases published in the literature showed that in all the cases, the diagnosis was confirmed only on histopathological examination (Table 1) [3-16].

**Table 1:** Review of previously published cases with Xanthogranulomatous osteomyelitis of appendicular skeleton IV, intravenous; CT, computed tomography; MRI, magnetic resonance imaging [3-16]

Authors	Year	Age/Sex	Site	Radiology Findings	Clinical and Radiological Diagnosis	Organisms grown and other findings	Treatment
Cozzutto et al.	1984	5/M 14/M	1^st^ rib; tibia	X-rays - osteolytic lesion; X-rays - mottled radiolucency	Ewings sarcoma and chronic osteomyelitis; chronic infection	—	En bloc resection of the first rib; excision of the lesion
Vankalakunti et al.	2007	50/F	Ulnar diaphysis	X-rays - Poorly defined osteolytic lesion	Tumor	Histiocytes positive for KP1, HAM56, CD11b, CD68	Curettage with bone grafting
Cennimo et al.	2009	41/M	Index finger and wrist	X-rays - Swelling of soft tissue MRI - abscess formation and synovial enhancement	Abscess formation with the enhancement of synovium	Mycobacterium marinum grown from culture	Antibiotics & Synovectomy
Kamat et al.	2011	13/M	Distal tibia	X-rays - Lytic lesion in the submetaphyseal region with sclerotic margin	Brodie’s abscess	Staphylococcus aureus	Curettage
Borjian et at.	2012	14/M	Humeral head; diaphysis of fibula	X-rays - Reaction in the periosteum and disruption of cortex CT; reaction in the periosteum and infiltration of bone marrow MRI signal abnormalities	Malignancy osteomyelitis	Staphylococcus aureus	Patient left hospital against medical advice
Nunes et al.	2012	56/M	Distal humeral metaphysis	Osteolytic lesion	Tumor	Histocytes positive for CD68	Curettage with bone grafting
Holmes et al.	2013	44/M	Distal tibia	Mass in the soft tissue	—	—	Curettage
Nalini et al.	2014	20/F	Femur (peritrochanteric region)	Osteolytic lesion with well-defined margins	—	—	Curettage with bone grafting
Rathi et al.	2014	50/M	Distal tibia	Osteolytic lesions with periosteal reaction	—	Pseudomonas grown from pus culture	IV antibiotics, arthrodesis
Sapra et al.	2015	34/M	Medial malleolus, talus, cuboid	Osteolytic lesions with marginal sclerosis	—	—	Curettage with bone grafting
Singh et al.	2015	65/F	Femur	Osteolytic lesions with well-defined margins	—	—	
Arul et al.	2016	20/M	Femur	Hyperintense lesion with a well-defined margin	—	—	Curettage
Baisakh et al.	2016	21/F	Distal epiphysis of femur; proximal metaphysis of tibia	Osteolytic lesions	—	—	—
Cheema et al.	2017	5/F	Humerus	Multiple osteolytic lesions	—	Non-typhus Salmonella	IV & oral antibiotics

Only three cases around the hip have been reported [10,13-14]. A relationship between bacterial infection and xanthogranulomatous inflammation has been determined in several organs such as kidneys and the gastrointestinal (GI) system but remains undetermined for bone [1,3-4]. Cultures have been positive for various organisms such as *Salmonella*, *S. aureus,* *Pseudomonas*, and *Mycobacterium marinum* [5-7,11]. In our reported case, the culture was positive for *S. aureus* and antibiotics were started accordingly.

The microscopic appearance of xanthogranulomatous inflammation on histology shows characteristic multinucleate giant cells interspersed with lipid-laden macrophages, which impart the characteristic yellow macroscopic appearance. This appearance, however, should be differentiated from a pseudoxanthomatous inflammation or malakoplakia characterized by Michaelis-Gutman bodies that stain positive with Von Kossa calcium and Prussian blue stains [17]. Differential diagnosis of Erdhiem-Chester disease radiologically shows osteosclerosis in almost all cases and histologically shows foamy histiocytes and fibrosis without neutrophilic infiltrations [18]. In cases of XO, neutrophilic infiltration is present with the bacterial infection. Langerhans’s cell histiocytosis shows eosinophilic infiltration and radiologically presents with lesions without surrounding sclerosis [19]. Infiltrative storage disorder can be differentiated from XO by clinical history, cardiac involvement, and histologically, foamy macrophages of the bone marrow with no signs of inflammation [20]. Metastatic cancer can be differentiated from XO with appropriate history and systemic signs.

Given the limited experience in dealing with this unusual presentation, there is no established therapeutic approach. Diagnosis becomes difficult mainly due to the lack of literature support. Treatment of XO is favorable when surgery is paired with antibiotics for which the culture is positive that remains the mainstay of treatment. The presentation of mimicking tuberculosis in our case can be explained by the fact that the disease was long-standing and the lytic lesions might have preceded the gross destruction of the joint. Although XO involving bone is rare, surgeons and pathologists should be aware that it can present as a lesion with the fluid collection and can have different variable presentations. 

## Conclusions

We would emphasize that any surgeon treating a case with a possible diagnosis of a metastatic, inflammatory condition, or infection-like tuberculosis but not responsive to the current line of treatment should always consider xanthogranulomatous inflammation. Although a rare condition, it can have diverse presentations, and hence, we affirm that histopathological examination remains the cornerstone for diagnosis. It can be associated with high- to low-grade infections that can mask the basic pathological picture. With tuberculosis, being a great mimicker of malignancy, uncommon bacterial infection, and even fungal infections, it would seem reasonable to also include xanthogranulomatous inflammation in the list of differential diagnoses.

## References

[1] Cozzutto C, Carbone A (1988). The xanthogranulomatous process: xanthogranulomatous inflammation. Pathol Res Pract.

[2] Nistal M, Gonzalez- Peramato P, Serrano A, Regadera J (2004). Xanthogranulomatous funiculitis and orchiepididymitis: report of 2 cases with immunohistochemical study and literature review. Arch Pathol Lab Med.

[3] Cozzutto C (1984). Xanthogranulomatous osteomyelitis. Arch Pathol lab Med.

[4] Vankalakunti M, Saikia UN, Mathew M, Kang M (2007). Xanthogranulomatous osteomyelitis of ulna mimicking neoplasm. World J Surg Oncol.

[5] Cennimo DJ, Agag R, Fleegler E (2009). Mycobacterium marinum hand infection in a “sushi chef”. Eplasty.

[6] Kamat G, Gramapurohit V, Myageri A, Shettar C (2011). Xanthogranulomatous osteomyelitis presenting as swelling in right tibia. Case Rep Pathol.

[7] Borjian A, Rezaei F, Eshaghi MA, Shemshaki H (2012). Xanthogranulomatous osteomyelitis. J Orthop Traumatol.

[8] Nunes R, Costa J, Martins M (2012). Osteomielite xantogranulomatosa do úmero. Rev Port Ortop Traum.

[9] Holmes BJ, Castelino-Prabhu S, Rosenthal DL, Ali SZ (2013). Xanthogranuloma of bone: a challenging imitator of malignancy. Acta Cytol.

[10] Nalini G (2014). Xanthogranulomatous osteomyelitis: a case report. Med J.

[11] Rathi M, Khattri J, Budania SK, Singh J, Awasthi S, Verma S (2014). Xanthogranulomatous osteomyelitis. Arch Med Health Sci.

[12] Sapra R, Jain P, Gupta S, Kumar R (2015). Multifocal bilateral xanthogranulomatous osteomyelitis. Indian J Orthop.

[13] Singh S, Batra S, Maini L, Gautam VK (2015). Xanthogranulomatous osteomyelitis of proximal femur masquerading as benign bone tumor. Am J Orthop.

[14] Arul P, Ramdas A, Varghese R, Kanchana B (2016). Xanthogranulomatous osteomyelitis of femur masquerading as neoplasm. Clin Cancer Investig J.

[15] Baisakh MR, Kar MR, Agrawal A, Mohapatra N (2016). Xanthogranulomatous osteomyelitis mimicking neoplasm: a rare entity. Indian J Pathol Microbiol.

[16] Cheema A, Arkader A, Pawel B (2017). Xanthogranulomatous osteomyelitis of the humerus in a pediatric patient with Alagille syndrome: a case report and literature review. Skeletal Radiol.

[17] Rauschkolb EN, Sandler CM, Patel S, Childs TL (1982). Computed tomography of renal inflammatory disease. J Comput Assist Tomogr.

[18] Yun EJ, Yeh BM, Yabes AP, Coakley FV, Kane CJ (2003). Erdheim-Chester disease: case report and review of associated urological, radiological and histological features. J Urol.

[19] Azouz EM, Saigal G, Rodriguez MM, Podda A (2005). Langerhans' cell histiocytosis: pathology, imaging and treatment of skeletal involvement. Pediatr Radiol.

[20] Ferreira CR, Gahl WA (2017). Lysosomal storage diseases. Transl Sci Rare Dis.

